# Diversity analysis of *Populus euphratica* endophytic bacteria in Tarim River Basin, China

**DOI:** 10.7717/peerj.15934

**Published:** 2023-08-28

**Authors:** Gang Cheng, Yan Cheng, Erkin Rahman

**Affiliations:** 1 Shangrao Normal University, College of Life Science, Shangrao, Jiangxi, China; 2 Xinjiang Academy of Environmental Protection Science, Xinjiang Key Laboratory for Environmental Pollution Monitoring and Risk Warning, Urumqi, Xinjiang, China; 3 Xinjiang University, College of Life Science and Technology, Urumqi, Xinjiang, China

**Keywords:** *Populus euphratica*, Endophytic bacteria, Phylogenetic analysis, Diversity

## Abstract

The bacterial diversity in *Populus euphratica* stem storage liquid samples grown in Shaya County and Yuli County of the Tarim River Basin was investigated. A culture-dependent (dilution spread plate method) and culture-independent method (PCR-RFLP technique) were used to identify the endophytic bacteria community structure and composition in *P. euphratica* in Tarim River Basin. Sixty-six bacterial strains were isolated from *P. euphratica* stem storage liquid samples on three agar media. The 16S rDNA gene was amplified and sequenced using bacterial universal primers. Phylogenetic analysis showed that the 66 strains belonged to three phyla (Firmicutes, Actinomycetes, and Gamma-Proteobacteria) and included 16 genera and 29 species. Among them, *Pseudomonas* (27.27%) and *Bacillus* (19.69%) were the dominant isolates. CGM-17 was a potentially new species of *Pantoea*. Restriction fragment length polymorphism of 16S rDNA gene amplified by polymerase chain reaction (PCR-RFLP) revealed 48 operational taxonomic units (OTUs). Phylogenetic analysis indicated that the 48 OTUs belonged to Firmicutes, Actinobacteria, Proteobacteria (α-, β-, γ-subgroup), Bacteroidetes, and Verrucomicrobia. Gamma-Proteobacteria was the dominant group, similarly to the culture-dependent method, accounting for 53% of the entire bacterial clone library. Our results indicate that *P. euphratica* endophytic bacteria diversity in the Tarim River Basin was rich, and the resources of endophytic bacteria were high. They provide valuable reference data and species resources for screening indigenous and functional strains of endophytic bacteria in *P. euphratica*.

## Introduction

Endophytes are non-pathogenic bacteria and fungi that can colonize the in or outside spaces of the cells of healthy plant tissues, establishing a symbiotic or alternate relationship with the host plant during the long-term evolution process (Hardoim, Van Overbeek & Van Elsas, 2008; Dai, Liu & Wang, 2014). At the same time, endophytic bacteria presence does not significantly change plants’ phenotypic characteristics and functions. They can directly or indirectly promote plant development by providing nutrients such as iron, phosphorus, and nitrogen and synthesizing hormones such as indole-3-acetic acid (IAA) and ethylene (Ahemad & Kibret, 2014; Santoyo et al., 2016). In addition, endophytic bacteria can also synthesize secondary metabolites with antimicrobial properties to induce host plant resistance. Therefore, they play a key role in biological nitrogen fixation, plant growth promotion, and plant biological disease control (Kim et al., 2016; Liotti et al., 2018).

*Populus euphratica* Oliv. is a large deciduous plant belonging to the family Salicaceae. It is a widespread and important tree species in the Tarim Basin in southern Xinjiang. *P. euphratica* has strong vitality and can tolerate extreme habitats in arid desert areas (Zhou et al., 2018). At present, the research on *P. euphratica* microbial diversity and composition focuses on soil microbial diversity (Bao et al., 2011; Chen et al., 2015; Liu et al., 2019; Wang et al., 2019), isolation and identification of culturable strains (Zilaygul et al., 2009; He et al., 2010). Furthermore, studies on endophytic bacteria of *P. euphratica* have mainly focused on the roots, stems, leaves, other tissues and organs, with only a few reports on the microbial diversity in the stem storage liquid (Yuan et al., 2007; Khayir et al., 2011; Maryam et al., 2011). The stem storage liquid of *P. euphratica* is a seasonal product in response to elevated temperatures and drought in summer. This liquid is stored in the trunk of *P. euphratica* and can objectively reflect its endophytic microorganisms’ diversity and community structure (Khayir et al., 2011; Hormathan et al., 2014). These results show that the *P. euphratica* forest is a unique and stable ecosystem in arid desert areas, rich in microbial species resources with a broad prospect for exploitation.

Currently, with the development of biochemistry and sequencing techniques, some new molecular biology techniques, such as PCR-RFLP techniques, DGGE analysis and metagenomic sequencing technologies, have brought great convenience to bacterial identification and microbial community structure (Saini et al., 2013; Beghalem et al., 2017; Fadiji & Babalola, 2020; Abid et al., 2022). PCR-RFLP technology has unique advantages over metagenomic sequencing technology. For example, PCR-RFLP technology is easier to use and requires less experimental expenses. Secondly, because bacterial 16S rDNA genes are of moderate size and contain both highly conserved sequences and considerable variation, PCR-RFLP analysis can better reflect differences between genera, species and closely related strains (Fadiji & Babalola, 2020). Therefore, the PCR-RFLP technique has been widely used by researchers to study bacterial diversity (Beghalem et al., 2017; Aravena et al., 2020).

By combining culture-dependent and culture-independent approaches, this study comprehensively analyzed the community structure and population diversity of *P. euphratica* endophytic bacteria in the middle reaches of the Tarim River Basin. It provides the basis for further discovery, characterization, and exploitation of functional microbial species resources.

## Materials and Methods

### Sampling

The sampling sites where the material was collected were the *P. euphratica* forests at the disused Achik River located in Shaya County (N40°56′, E82°15′) and at the disused Ugan River located in Yuli County (N41°00′, E85°04′), Xinjiang Province, China. These forests are characterized by high density, flourishing growth, and low degradation degree and are sparsely populated. They are rare natural *P. euphratica* forests not affected by human activities. A total of eight *P. euphratica* trees in the two sample sites were selected to obtain samples. At each sampling site, four individual *P. euphratica* trees (*P. euphratica* tree height ≥6 m and DBH ≥50 cm) were randomly selected. Tree height was measured using a tree height gauge (Vertex-IV, Haglof, Dalarna, Sweden) and a tape measure for diameter at breast height. A sterilized annular ring drill was used to drill holes in the sterilized *P. euphratica* trunk, the hole approximately 1.6 m above the ground. The *P. euphratica* stem storage liquid was collected in sterilized centrifuge tubes, which were stored at 4 °C for further analysis. The borehole on the *P. euphratica* trunk was filled with specially prepared *P. euphratica* stakes to prevent the *P. euphratica* tree from dying (Khayir et al., 2011).

### Isolation and purification of culturable strains

Culturable strains were isolated using three types of culture media: LB, King B, and NA. The Luria-Bertani medium (LB) contained 1% peptone, 1% NaCl, 0.5% yeast extract, at a pH of 7.5–8.0. The King B medium (King B) contained 2% peptone, 0.03% K_2_HPO_4_, 0.15% MgSO_4_·7H_2_O, 1.5% (v/v) glycerin, at a pH of 7.5–8.0. Nutrient Agar medium (NA) containing 3% beef extract, 0.5% peptone, 0.5% NaCl, at a pH of 7.5–8.0.

The storage liquid collected from 8 *P. euphratica* trees was mixed in an equal amount, gradient diluted with a liquid medium. It was subsequently spread on the LB, King B, and NA solid medium surface, then cultured upside down for 3–7 days at 37 °C. Single colonies with significant differences in morphology were selected, repeating purification 2–3 times. The purified bacterial strains were collected in a liquid medium containing 40% (v/v) glycerin and stored at −80 °C.

### Evaluation of culture medium predominant index and clone libraries

The culture medium predominant index was calculated by the formula: D = N/NT, where N was the number of endophytic bacteria species isolated from the medium, and NT was the total number of isolated strains (Lan et al., 2008).

The clone library was evaluated using two methods: (1) Coverage value. The formula C = 1−nl/N was used, where nl represents the number of OTUs that only appeared once in the clone library, and N represents the total number of clones in the library. The C value theoretically represents the proportion of microbial species in the clone library to all microbial species in the sample. (2) Rarefaction curves. Rarefaction curves were calculated using Estimates 8.0 software (http://viceroy.eeb.uconn.edu/estimates).

### Total DNA extraction and bacterial 16S rRNA gene amplification

The genomic DNA of culturable bacterial strains was extracted using the sodium dodecyl sulfate-proteinase K-cetyltrimethylammonium bromide (CTAB) method (Allen et al., 2006). PCR amplifications were carried out targeting the 16S rRNA gene with the 27F (5′-AGAGTTTGATCACTGGCTCAG-3′) and 1492R (5′-TACGGYTACCTTGTTACGACTT-3′) primers (Zhang et al., 2021).

The PCR reaction mixture (50 µL) contained DNA template 100 ng, 1× Taq reaction buffer (R001A; TaKaRa, Beijing, China), 200 μmol dNTPs, 10 pmol of each P1 and P2 PCR primers, and 1.25 U Taq DNA polymerase (R001A; TaKaRa, Beijing, China). Thermal cycling conditions were as follows: an initial denaturation at 94 °C for 5 min, followed by 30 cycles of 94 °C for 30 s, annealing at 55 °C (27F and 1492R)/53 °C (799F and 1492R) for 30 s, 72 °C for 1 min, with a final extension at 72 °C for 8 min. PCR products were visualized by 1 % agarose gel electrophoresis and purified using the TIANgel Midi Purification Kit (DP209; Tiangen, Beijing, China) as described by the manufacturer. The purified PCR amplicons of culturable strains were sent to Sangon Biotech (Shanghai) Co., Ltd. for sequencing.

The purified PCR products from the total DNA extracted from the *P. euphratica* storage liquid were inserted into the pMD18-T vector by overnight incubation in a water bath at 16 °C. They were subsequently transformed into DH5α competent cells (CB101; Tiangen, Beijing, China). Positive clones were screened for standard blue and white screening. Colonies randomly picked were screened directly for inserts by performing colony PCR. All positive clones were collected in a Luria-Bertani liquid medium containing 40 % (v/v) glycerin and stored at −80 °C.

### Culture-independent PCR-RFLP bacterial community analysis

For the culture-independent approach, the total DNA was extracted as previously reported with some modifications (Krsek & Wellington, 1999). The primer pair 799F (5′-AACAGGATTAGATACCCTG-3′) and 1492R (5′-GGTTACCTTGTTACGACTT-3′) were used to amplify the DNA of *P. euphratica* endophytic bacteria (Sun et al., 2008). These primers do not amplify *P. euphratica* chloroplast DNA. PCR amplification with 799F and 1492R results in an amplicon of approximately 735 bp.

PCR-RFLP was performed to analyze the diversity of positive clones. The PCR reaction mixture (25 µL) contained 2 µL *E. coli* carrying the cloned insert DNA, 1× Taq reaction buffer (R001A; TaKaRa, Beijing, China), 200 μmol dNTP, 10 pmol of each M13–47 and M13–48 PCR primers, and 0.75 U Taq DNA polymerase (R001A; TaKaRa, Beijing, China). Thermal cycling conditions were as follows: an initial denaturation at 94 °C for 5 min, followed by 35 cycles of 94 °C for 30 s, annealing at 56 °C for 30 s, 72 °C for 1 min, with a final extension at 72 °C for 8 min.

The PCR product digestion was performed by modifying the Miao et al. (2013) method: 20 µL of the PCR products was digested using the restriction enzyme *Hae* III for 12 h at 37 °C, the restriction fragments were separated on a 2% agarose gel running in 1× TAE buffer at 100 V for approximately 40 min. Clones with different enzyme digestion patterns were selected and grouped into OTUs, as described by Aravena et al. (2020). Subsequently, the selected OTUs were sent to Sangon Biotech (Shanghai) Co., Ltd. for 16S rRNA gene sequencing.

### Phylogenetic analysis and bacterial distribution analysis

The sequences obtained were compared and analyzed using EZTAXON (EzTaxon server 2.1) and BLAST (http://www.ncbi.nlm.nib.gov/blast/blast.cgi). The related species 16S rDNA sequences were retrieved from the NCBI nucleotide database. MEGA 7.0 software was used for the construction of phylogenetic trees. Bootstrap analysis was performed on 1,000 random samples taken from the multiple sequence alignment analysis and was carried out using Clustal W. Phylogenetic analysis was performed by the Neighbor-Joining method (Kumar, Stecher & Tamura, 2016).

The bacterial community structure was analyzed using the Venny 2.1 software (https://bioinfogp.cnb.csic.es/tools/venny/index.html).

## Results

### Isolation characteristics of endophytic bacteria in the culture medium

The *P. euphratica* stem storage liquid endophytic bacteria isolated by the LB, NA and King B culture medium were different (Table 1). A total of 66 strains of bacteria were isolated by the culture-dependent approach. By observing colony characteristics and cell morphology, it was found that the LB medium had the largest number of colonies and rich colony morphology. In contrast, King B medium had a smaller colony number and a single colony morphology.

**Table 1:** The diversity and dominant index of endophyte bateria on three types of different medium (LB, NA and King B).

Medium	pH	Genus	Species	Strain	Dominant index
LB	7.5–8	10	17	29	0.567
NA	7.5–8	6	10	24	0.333
King B	7.5–8	5	7	13	0.233

The culture medium significantly affected the number and type of bacterial strains isolated. A total of 29 strains of endophytic bacteria were isolated from LB medium, belonging to 10 genera. A total of 24 strains were isolated from the NA medium, belonging to six genera, and 13 strains were isolated from the King B medium, belonging to five genera. There were no common strains in the three medium, among which NA medium and LB medium had three common strains: *Pseudomonas xinjiangensis*, *Brenneria salicis* and *Bacillus safensis*. However, NA medium and King B medium had only one common strain, *Erwinia toletana*. LB medium and King B medium had no common strains.

Moreover, according to the dominance index in each of the three media, the dominance index of LB medium was 0.567, and that of NA medium and King B medium was 0.333 and 0.233, respectively. The above results indicated that the endophytic bacteria diversity isolated from the LB medium was the highest, while the King B medium had the lowest diversity.

### Identification of endophytic bacteria in *Populus euphratica* based on 16S rDNA sequences

In this study, a total of 66 endophytic bacteria strains were obtained by the culture-dependent approach. Their 16S rDNA amplified fragments were sequenced, and the sequencing results were submitted to GenBank. The accession numbers of the isolates 16S rDNA sequences were JQ353770–JQ353835. The similarity comparison results are shown in Table 2. The sequencing data indicated that the 66 strains of culturable endophytic bacteria belonged to three major phylogenetic groups, Firmicutes, Actinobacteria, and Gamma-Proteobacteria, with 30 species belonging to 16 genera.

**Table 2:** 16S rDNA sequences similarity analysis of isolated endophytic bacteria by culture-dependent method.

Isolate/OTUs	Phylum	Nearest type strain (accession No.)	Similarity (%)	Isolate/OTUs (accession No.)
CBN-33	Proteobacteria	*Pseudomonas sabulinigri* (KC842266.1)	99.78	JQ353783
CGN-9		*Pseudomonas sabulinigri* (KC842266.1)	100.00	JQ353832
CGN-3		*Pseudomonas sabulinigri* (KC842266.1)	99.93	JQ353828
CBN-21		*Pseudomonas xinjiangensis* (KJ210647.1)	99.71	JQ353781
CBN-5		*Pseudomonas xinjiangensis* (KF843721.1)	99.42	JQ353772
CBN-18		*Pseudomonas xinjiangensis* (KF843721.1)	99.57	JQ353779
CBN-6-2		*Pseudomonas xinjiangensis* (KJ210647.1)	99.78	JQ353774
CBN-32		*Pseudomonas pelagia* (KF817700.1)	100.00	JQ353782
CBN-11-2		*Pseudomonas xinjiangensis* (KJ210647.1)	100.00	JQ353777
CGN-2		*Pseudomonas xinjiangensis* (KJ210647.1)	100.00	JQ353827
CGN-18		*Pseudomonas xinjiangensis* (KJ210647.1)	99.93	JQ353835
CGN-17		*Pseudomonas xinjiangensis* (KJ210647.1)	99.93	JQ353834
CGN-13		*Pseudomonas xinjiangensis* (KJ210647.1)	99.93	JQ353833
CGL-10		*Pseudomonas xinjiangensis* (KF843721.1)	99.64	JQ353804
CGL-22		*Pseudomonas xinjiangensis* (KJ210647.1)	99.93	JQ353803
CGL-13		*Pseudomonas xinjiangensis* (KJ210647.1)	99.93	JQ353796
CGL-2		*Pseudomonas xinjiangensis* (KJ210647.1)	100.00	JQ353784
CBN-1		*Pseudomonas luteola* (MH281751.1)	99.19	JQ353770
CBN-17		*Erwinia billingiae* (KM891551.1)	98.24	JQ353778
CBN-3		*Erwinia toletana* (AF130963)	98.15	JQ353771
CBN-10		*Erwinia toletana* (AF130963)	98.22	JQ353776
CGM-1		*Erwinia toletana* (AF130963)	98.15	JQ353805
CGM-6		*Erwinia toletana* (AF130963)	98.22	JQ353810
CBN-19		*Brenneria salicis* (KC840819.1)	99.56	JQ353780
CGL-12		*Brenneria salicis* (KJ210646.1)	98.82	JQ353795
CGL-5		*Brenneria salicis* (KJ210646.1)	99.93	JQ353787
CGN-4		*Brenneria salicis* (KJ210646.1)	99.93	JQ353829
CGM-5		*Enterobacter cloacae* (MN594805.1)	99.71	JQ353809
CGM-4		*Enterobacter cloacae* (MN594805.1)	99.78	JQ353808
CGM-3		*Enterobacter cloacae* (MN594805.1)	99.56	JQ353807
CGM-2		*Enterobacter cloacae* (MN594805.1)	99.71	JQ353806
CGM-15		*Raoultella terrigena* (OM200145.1)	98.56	JQ353815
CGM-14		*Raoultella terrigena* (OM200145.1)	98.14	JQ353814
CGM-8		*Raoultella terrigena* (OM533593.1)	98.42	JQ353812
CGM-11		*Pantoea wallisii* (JF295057)	98.06	JQ353813
CGM-17		*Pantoea rwandensis* (JF295055)	96.01	JQ353817
CGL-21		*Halomonas meridiana* (KC843375.1)	98.00	JQ353802
CG-8		*Acinetobacter lwoffii* (MN704528.1)	99.34	JQ353818
CGL-18	Firmicutes	*Bacillus tequilensis* (OM980075.1)	100.00	JQ353801
CGL-9		*Bacillus tequilensis* (OM980075.1)	99.93	JQ353792
CGL-8-1		*Bacillus tequilensis* (OM980075.1)	99.93	JQ353790
CBN-8		*Bacillus safensis* (MT378374.1)	99.71	JQ353775
CGL-16-2		*Bacillus safensis* (MT642941.1)	100.00	JQ353800
CGL-3		*Bacillus safensis* (MT642941.1)	99.93	JQ353785
CGN-8		*Bacillus safensis* (KT728849.1)	99.93	JQ353831
CG-30		*Bacillus pumilus* (MT071175.1)	99.72	JQ353824
CG-26		*Bacillus pumilus* (MN551170.1)	98.45	JQ353823
CG-15		*Bacillus pumilus* (MN581190.1)	99.65	JQ353821
CG-9		*Bacillus licheniformis* (JF798392.1)	96.29	JQ353819
CG-11		*Bacillus anthracis* (MF101005.1)	96.19	JQ353820
CG-20		*Bacillus anthracis* (KF779074.1)	98.94	JQ353822
CGL-16-1		*Planococcus antarcticus* (OK299055.1)	99.29	JQ353799
CGL-15		*Planococcus antarcticus* (AJ314745)	97.59	JQ353798
CGL-8-2		*Planococcus salinarum* (KF177264.1)	99.78	JQ353791
CGL-6		*Planococcus salinarum* (KF177264.1)	99.71	JQ353788
CGN-7		*Planococcus maitriensis* (KC843409.1)	99.93	JQ353830
CGN-1		*Planococcus maitriensis* (KC843409.1)	99.93	JQ353826
CGL-7		*Planococcus rifietoensis* (KF749392.1)	99.21	JQ353789
CGL-4		*Planomicrobium okeanokoites* (KF749414.1)	98.08	JQ353786
CGL-14		*Planomicrobium chinense* (KC842238.1)	99.86	JQ353797
CBN-6-1		*Oceanobacillus manasiensis* (MT125837.1)	99.79	JQ353773
CG-46		*Brevibacterium halotolerans* (AM747812)	99.93	JQ353825
CGM-16	Actinobacteria	*Cellulomonas hominis* (KP282823.1)	99.86	JQ353816
CGM-7		*Cellulomonas terrae* (NR_043257.1)	98.29	JQ353811
CGL-11		*Nesterenkonia aethiopica* (AY574575)	98.10	JQ353794
CGL-10-2		*Agrococcus citreus* (MK318614.1)	99.93	JQ353793

Among the 66 endophytic bacteria, 38 strains, or 57.58 % of the total, belonged to Gamma-Proteobacteria, thus being the most dominant group. These strains belonged to eight known genera. Pseudomonas was the dominant strain, with 18 strains accounting for 27.27 % of the total isolated strains. A total of 14 strains had a high similarity of sequences, ranging from 99.42% to 100%, with *Pseudomonas xinjiangensis* model strains. It indicated that these 14 strains were different mutants of the same strain, which showed these strains have more multi-directional dissimilation tendency and high detection frequency. They were the dominant species of culturable endophytic bacteria in this study. Interestingly, the taxonomic unit of *Pseudomonas luteola* represented by CBN-1 (JQ353770) was not previously identified in *P. euphratica* stem storage liquid samples (Khayir et al., 2011; Hormathan et al., 2014). The sequence similarity between CGM-17 (JQ353817) and the model strain *Pantoea rwandensis* (JF295055) was only 96.01%, indicating that CGM-17 was a potential new species.

There were 24 strains belonging to Firmicutes, the second most dominant group accounting for 36.36% of the total isolated strains. These strains belonged to five known genera. *Bacillus* was the second most dominant genus, accounting for 19.69 % of the isolated strains. A total of 13 *Bacillus* strains were isolated, belonging to 5 taxonomic units. The taxonomic unit of *Bacillus licheniformis* represented by CG-9 (JQ353819) was firstly found in the stem storage liquid samples of *P. euphratica* in our study, and its sequence similarity with model strain *Bacillus licheniformis* (JF798392.1) was only 96.29 %. In addition, the sequence similarity between CG-11 (JQ353820) and model strain *Bacillus anthracis* (MF101005.1) was only 96.19 %, speculating that CG-9 and CG-11 were potential new species.

Four endophytic bacteria strains belonging to four known genera of *Actinobacteria*, *Nesterenkonia*, *Agrococcus*, and *Cellulomonas*, were identified, accounting for only 6.06% of the total number of isolates. In addition, 14 other genera of endophytic bacteria were isolated, which fully reflected the community diversity of *P. euphratica* stem storage liquid samples. By comparing the previous study’s *P. euphratica* stem storage liquid samples (Khayir et al., 2011; Hormathan et al., 2014), it was found that among the 14 genera, *Erwinia*, *Raoultella*, *Agrococcus*, and *Cellulomonas* were isolated from stem storage liquid for the first time. Similarly, the species *Pantoea wallisii*, *Pantoea rwandensis*, and *Oceanobacillus manasiensis* were for the first time isolated from *P. euphratica* located in Tarim River Basin.

**Figure 1:**
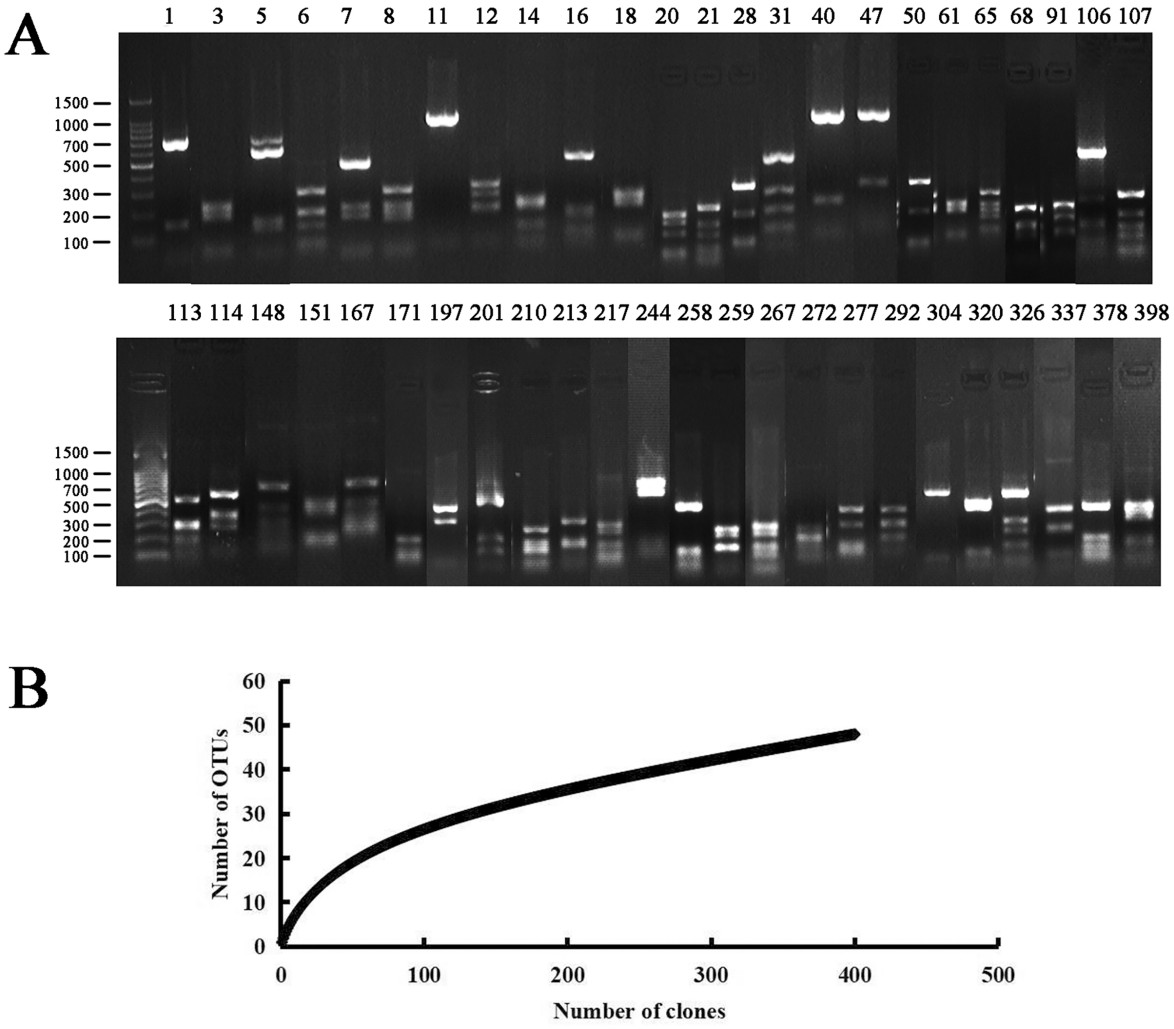
PCR-RFLP analysis of the endophytic bacterial positive clone. (A) Lanes 1 to 398 correspond to all 48 restriction patterns of the positive clones from the identified species digested with *Hae* III. Molecular weight (MW): 100 bp DNA Ladder (Takara). (B) Rarefaction curves of endophytic bacterial 16S rRNA gene clone library from *Populus euphratica* in Tarim River Basin. The bacterial clone library coverage rate was: C = 94%, when the number of clones was more than 300, the upward trend of the curve slowed down.

The bacterial clone library was obtained by the culture-independent method. A total of 48 OTUs (400 clones in total) were obtained after RFLP sequencing (Fig. 1). The sequence accession numbers were JQ941396–JQ941443, and the similarity comparison results are shown in Table 3.

**Table 3:** 16S rDNA sequences similarity analysis of isolated endophytic bacteria by culture-independent method.

Isolate/OTUs	Phylum	Nearest type strain (accession No.)	Similarity (%)	Isolate/OTUs (accession No.)
xj-61	Proteobacteria	*Pseudomonas xinjiangensis* (HQ696425.1)	99.85	JQ941414
xj-3		*Pseudomonas populi* (NR_148798.1)	99.44	JQ941397
xj-8		*Brenneria salicis* (NR_114714.1)	98.62	JQ941401
xj-14		*Brenneria salicis* (HM441252.1)	98.47	JQ941404
xj-259		*Rhodobaca bogoriensis* (NR025089.1)	96.89	JQ941433
xj-267		*Seohaeicola saemankumensis* (MK493559.1)	99.29	JQ941434
xj-12		*Sphingobium xanthum* (NR_133860.1)	99.72	JQ941403
xj-197		*Azoarcus* sp. (AF011329.1)	97.53	JQ941426
xj-320		Uncultured bacterium gene (AB355045.1)	99.44	JQ941439
xj-31		Uncultured gamma proteobacterium clone ZLL-D58 (JF806933.1)	98.62	JQ941410
xj-337		Uncultured bacterium clone SINP718 (HM127665.1)	99.86	JQ941441
xj-40	Firmicutes	*Alloiococcus otitis* (AY957475.1)	96.42	JQ941411
xj-47		*Alkalibacterium olivapovliticus* (NR_112658.1)	99.59	JQ941412
xj-106		*Alkalibacterium olivapovliticus* (NR_112658.1)	99.03	JQ941418
xj-304		*Alloiococcus otitis* (AY957475.1)	96.56	JQ941438
xj-201		*Carnobacterium mobile* (LT223645.1)	97.23	JQ941427
xj-5		*Carnobacterium mobile* (LT223645.1)	97.09	JQ941398
xj-292		*Carnobacterium mobile* (NR_040926.1)	97.11	JQ941437
xj-28		*Clostridium tagluense* (NR_043698.1)	97.80	JQ941409
xj-277		*Clostridium bowmanii* (NR_114879.1)	99.44	JQ941436
xj-50		*Clostridium bowmanii* (NR_114879.1)	99.31	JQ941413
xj-21		*Thermacetogenium phaeum* (NR_074723.1)	90.99	JQ941408
xj-1		*Marinilactibacillus piezotolerans* (MN636689.1)	99.58	JQ941396
xj-7		*Planococcus antioxidans* (KU601236.2)	100.00	JQ941400
xj-244		*Lachnotalea glycerini* (MF953294.1)	95.74	JQ941431
xj-378		*Clostridium bowmanii* (NR_114879.1)	99.30	JQ941442
xj-91		Uncultured bacterium gene (AB514660.1)	97.09	JQ941417
xj-16		Uncultured bacterium clone PeHg66 (FJ374225.1)	93.20	JQ941405
xj-258		Uncultured bacterium clone Rc601 (JQ617858.1)	97.79	JQ941432
xj-323		*Candidatus Syntropholuna* (KU681304.1)	96.71	JQ941440
xj-114		Uncultured bacterium (FN985268.1)	91.86	JQ941421
xj-88		Uncultured bacterium clone MFC-CL-38 (JN967069.1)	99.04	JQ941416
xj-11		*Eisenbergiella tayi* (LC515631.1)	95.35	JQ941402
xj-107	Actinobacteria	*Cellulomonas* sp. (EU303275.1)	98.61	JQ941419
xj-272		*Arthrobacter agilis* (KF306343.1)	99.86	JQ941435
xj-148		*Demequina sediminis* (NR_158021.1)	99.72	JQ941422
xj-213	Bacteroidetes	*Litoribacter populi* (MN209790.1)	96.77	JQ941429
xj-167		*Bacteroides* sp. (AY554420.1)	96.73	JQ941424
xj-18		*Bacteroides* sp. (AY554420.1)	96.40	JQ941406
xj-113		*Flavobacterium alkalisoli* (MN310902.1)	96.36	JQ941420
xj-20		*Planktosalinus lacus* (NR_149250.1)	98.31	JQ941407
xj-151		*Rumen bacterium* (HQ640508.1)	96.34	JQ941423
xj-6		*Ruminobacillus xylanolyticum* (DQ178248.1)	96.61	JQ941399
xj-65		*Planktosalinus lacus* (NR_149250.1)	98.32	JQ941415
xj-171		Uncultured bacterium clone Inoculum14 (HM008286.1)	96.51	JQ941425
xj-210		*Mariniphaga anaerophila* (NR_134076.1)	98.11	JQ941428
xj-217		Uncultured bacterium (FN985279.1)	96.37	JQ941430
xj-398	Verrucomicrobia	Uncultured Verrucomicrobia bacterium clone (HQ857650.1)	100.00	JQ941443

Phylogenetic analysis and RDP classification indicated that the 48 OTUs isolated from the clone library belonged to Firmicutes, Actinobacteria, Alpha-Proteobacteria, Beta-Proteobacteria, Gamma-Proteobacteria, Bacteroidetes, and Verrucomicrobia. Proteobacteria was the dominant group, accounting for 59.75% of the total OTUs. Among the three subgroups, Alpha-Proteobacteria accounted for 6.5%, Beta-Proteobacteria accounted for 0.5%, and Gamma-Proteobacteria accounted for 52.75% of the total OTUs. Firmicutes, the second most dominant group, accounted for 28.25% of the total OTUs. The third most dominant group was Bacteroidetes, accounting for 10.25% of the total OTUs. The other phyla accounted for a smaller percent abundance (Table 3).

### 16S rDNA sequence phylogenetic analysis of endophytic bacteria in *Populus euphratica*

**Figure 2:**
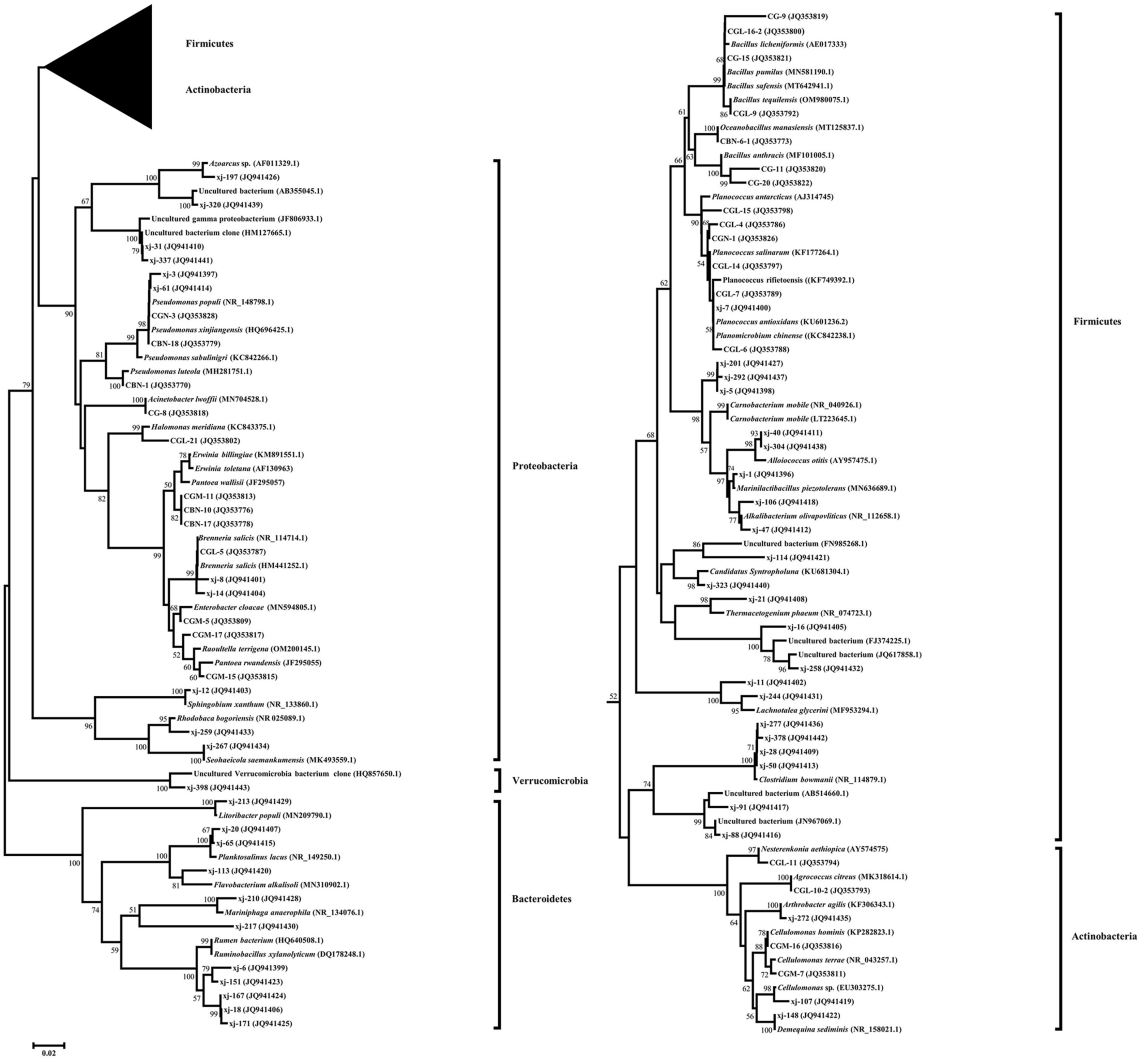
Phylogenetic tree of endophytic bacteria isolates from *Populus euphratica* based on the partial 16S rDNA sequences. The phylogenetic tree was constructed using the Neighbor-Joining method. Accession numbers indicated after species names sequences are from GenBank. The isolates numbered xj- were clones isolated by the culture-independent method, and the other strains were isolated by the culture-dependent method. Bootstrap analysis was carried out with 1,000 replicates. Branches corresponding to partitions reproduced in less than 50% of bootstrap replicates are collapsed. Bootstrap values of the branches are indicated on a 0.02 basis. Evolutionary analyses were conducted in MEGA 7.0.

A phylogenetic tree fusing the partial 16S rDNA sequences was constructed using the neighbor-joining method (Fig. 2). Five phylogenetic groups, Firmicutes, Actinobacteria, Proteobacteria, Bacteroidetes, and Verrucomicrobia, covered all taxonomic units of the bacteria identified in *P. euphratica* stem storage liquid in the Tarim River Basin.

Among them, 66 endophytic bacteria obtained from the culture-dependent approach were clustered into Firmicutes, Actinobacteria, and Proteobacteria. Gamma-Proteobacteria was the most dominant group of culturable endophytic bacteria clustered in a large branch of the phylogenetic tree. Firmicutes were the second most dominant group, and the 24 isolates were clustered on a large branch. *Bacillus* (19.69%) was the second most dominant genus, in agreement with the reported endophytic bacteria literature (Khayir et al., 2011; Ju, Ouyang & Zhang, 2014). Only four endophytic bacteria strains belonged to Actinobacteria, and their evolutionary branch was divided further into three branches. CGL-11 (JQ353794) and clone xj-272 (JQ941435) were clustered in the same clade, while the sequence similarity between CGL-11 (JQ353794) and *Nesterenkonia aethiopica* (AY574575) was 98.10%.

Proteobacteria was the most dominant group in the bacterial clone library obtained by the culture-independent method and included 11 OTUs. Its evolutionary branches were divided into three clades. Alpha-Proteobacteria and Beta-Proteobacteria were unique to the culture-independent method, clustering on separate clades. The clones and isolates of Gamma-Proteobacteria, the dominant group in the phylum, were clustered on a large branch. The sequence similarity between the dominant clone xj-14 (JQ941404), accounting for 20.25% of the total clone library of the culture-independent method, and model strain *Brenneria salicis* (HM441252.1) was 98.47%. The clones belonging to the genus *Erwinia* and *Pseudomonas* followed in abundance, accounting for 19.5% and 7% of the total bacterial clone library, respectively.

Firmicutes were the second most dominant group in the bacterial clone library, accounting for 22 OTUs. The sequence similarity between the dominant clone xj-40 (JQ941411), which accounted for 5.75 % of the total bacterial clone library, and the model strain *Alloiococcus otitis* (AY957475.1) was 96.42%. Clone xj-323 (JQ941440) alone occupied a taxon alone and had 96.71% sequence similarity with *Candidatus Syntropholuna* (KU681304.1). On the other hand, the sequence similarity between clone xj-21 (JQ941408) and model strain *Thermacetogenium phaeum* (NR_074723.1) was only 90.99%.

Bacteroidetes and Verrucomicrobia were only found in the culture-independent method. Bacteroidetes was the third most dominant group, with 11 OTUs, accounting for 10.25% of the total bacterial clone library. Its evolutionary branch was further divided into three branches. The clone xj-213 (JQ941429), which formed its own branch, had a 96.77% sequence similarity with *Litoribacter populi* (MN209790.1).

Actinobacteria was the fourth most dominant group in the bacterial clone library, with three OTUs, accounting for 1.5% of the total bacterial clone library. The clone xj-107 (JQ941419) and the culturable strains CGM-7 (JQ353811) and CGM-16 (JQ353816) were clustered in the same clade, classified as *Cellulomonas*. Verrucomicrobia contained only one OTU. Clone xj-398 (JQ941443) occupied a taxon alone and had 100% sequence similarity with an Uncultured Verrucomicrobia bacterium (HQ857650.1).

### Analysis of bacterial species distribution in the stem storage liquid of *P. euphratica*

**Figure 3:**
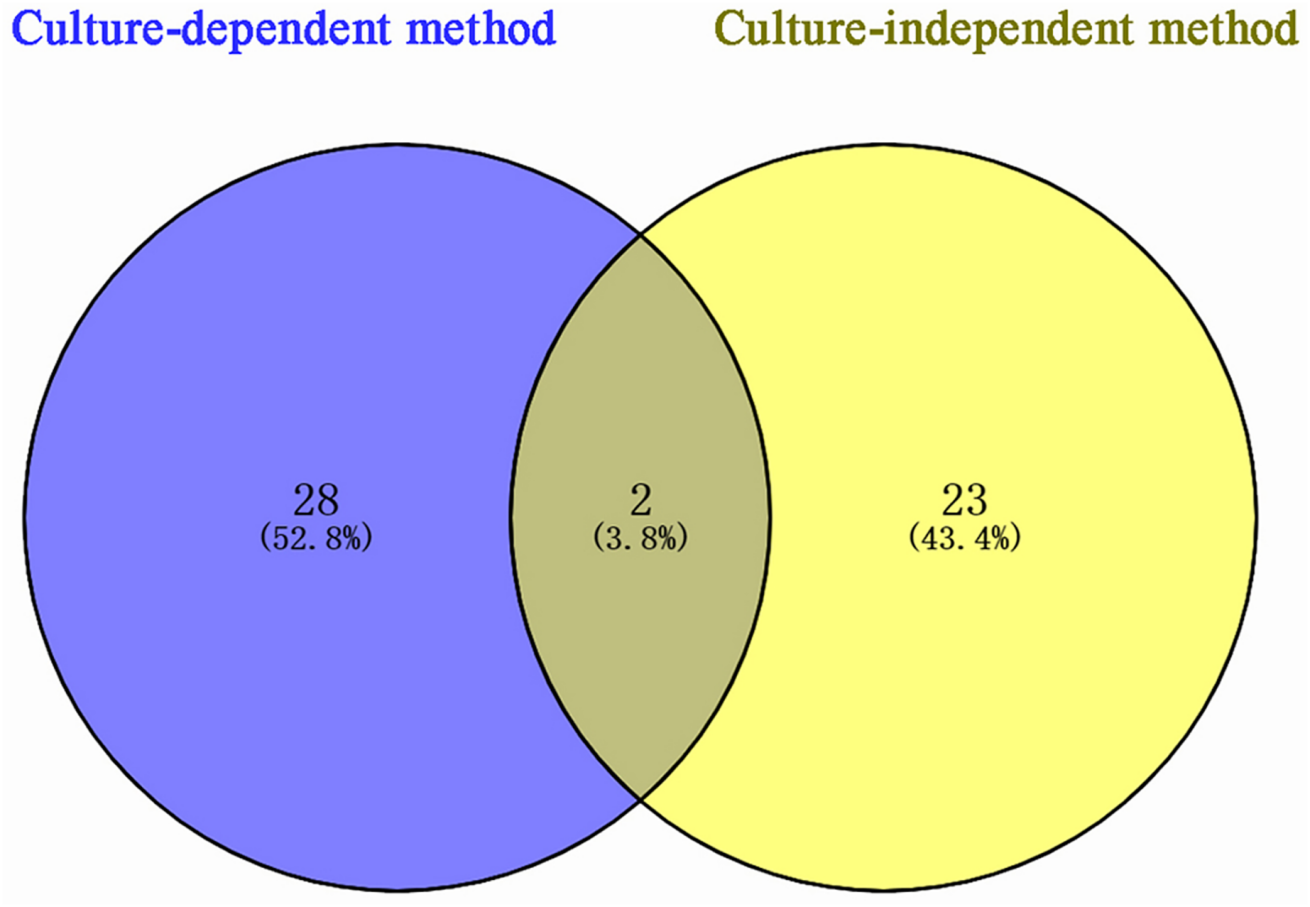
Venn diagram of *Populus euphratica* endophytic bacteria in Tarim River Basin. Different methods of strain isolation are shown in different colors, and the Numbers in the figure represent the number of specific or common components. A total of two species of overlapping endophytic bacteria can be shown intuitively, *Brenneria salicis* and *Pseudomonas xinjiangensis*, respectively.

According to sequencing results, 53 bacterial species were identified in *P. euphratica* stem storage liquid, of which 30 species were isolated by the culture-dependent method and 25 species were identified by the culture-independent method (Fig. 3). The two methods exhibited obvious differences in the distribution of bacterial community structure. Only *Brenneria salicis* and *Pseudomonas xinjiangensis* were isolated by both methods, while most of the bacterial species were uniquely identified in the respective methods. Among the 30 species of bacteria obtained by the culture-dependent, 28 species (52.8% of the total identified) were unique. Thus, among the 25 species of bacterial OTUs identified by the culture-independent method, 23 species (43.4% of the total identified) were unique. These results indicate that the different isolation and identification approaches could be combined and compensate each other for the accurate bacterial community structure determination in the stem storage liquid samples of *P. euphratica*.

## Discussion

In this study, a total of 66 culturable strains were isolated from the stem storage liquid samples of *P. euphratica* by the culture-dependent method, belonging to 30 taxa, 16 genera, and 3 phyla. Additionally, 48 OTUs were obtained by the culture-independent method, belonging to 5 phyla. The phyla Firmicutes, Actinobacteria, and Gamma-Proteobacteria, were shared by the two methods. The diversity of endophytic bacteria isolated by the culture-dependent method differed significantly in the three different culture mediums used. The dominance index of the LB medium was 0.567, the NA medium was 0.333, and the King B medium was 0.233. In addition, strains of *Erwinia*, *Raoultella*, *Abrococcus*, and *Cellulomonas* were isolated and cultured from the *P. euphratica* stem storage liquid samples in Tarim River Basin for the first time (Khayir et al., 2011; Hormathan et al., 2014), indicating the utility of selective culture media.

Gamma-Proteobacteria was the dominant group of culturable bacteria, accounting for 57.58% of the total culturable bacteria in the stem storage liquid of *P. euphratica*. *Pseudomonas* was the second most dominant group, accounting for 27.27% of the isolates, followed by *Bacillus*, with 19.69%. In the culture-independent method, the dominant group was similarly Gamma-Proteobacteria, accounting for 52.75% of the bacterial clone library. The dominant clones belonged to the genus *Brenneria*at 20.25%. Secondly, Gamma-Proteobacteria strains have been previously found frequently in the storage liquid samples of *P. euphratica* (Khayir et al., 2011; Ju, Ouyang & Zhang, 2014). In our study, the dominant species of culturable bacteria was *Pseudomonas xinjiangensis*, accounting for 21.21% of the isolated strains. Notably, the clones xj-3 (JQ941397) and xj-61 (JQ941414) found in the culture-independent method also belonged to Pseudomonadaceae after RDP classification, accounting for 12.25% of the bacterial clone library.

The *P. euphratica* endophytic bacteria diversity is rich, with numerous strains with biocontrol functions, such as plant growth-promoting bacteria (PGPB). Compared with the traditional culture-dependent method, the endophytic bacteria obtained by the culture-independent method are more numerous in terms of species count. Compared with the results of culturable endophytic bacteria in *P. euphratica* stem storage liquid (Khayir et al., 2011; Hormathan et al., 2014), Verrucomicrobia was identified for the first time in the screening of *P. euphratica* endophytic bacteria clones. In terms of genera, only *Brenneria*, *Planococcus*, *Planomicrobium*, *Pseudomonas*, and *Marinilactibacillus* were common between the two methods. Secondly, some bacterial groups that were identified in the culture-dependent method could not be observed in the culture-independent method. *Bacillus* was the dominant genus in the endophytic bacterial community in the culturable endophytic bacteria in *P. euphratica* stem storage liquid. However, it was not identified in the culture-independent method.

Among them, certain *Brenneria*, *Erwinia*, and *Pseudomonas* strains are plant disease-related bacteria, including pathogenic and non-pathogenic strains. It has been previously reported that *Erwinia Billingiae* may have antagonistic functions toward fire blight. Thus, it can be exploited for its biocontrol (Kube et al., 2010). Moreover, *Cellulomonas* strains are widely distributed in the soil and in rotten vegetables, and it has been reported that they can promote the growth of plant roots and new shoots (Egamberdiyeva & Höflich, 2002). Certain strains of *Bacillus* and *Arthrobacter* have been reported to increase the proline content in plants, which can maintain the pH and some enzyme activities in cells, thus helping plants resist salt stress (Michiels, Croes & Vanderleyd, 1991). Strains of *Acinetobacter* isolated from *P. euphratica* leaves were shown to promote salt tolerance of wheat seedlings (Ju, Ouyang & Zhang, 2014).

In addition, the detection of new species in *P. euphratica* from the stem storage liquid was high. The sequence similarity of culturable bacteria strains CGM-17 (JQ353817), CG-9 (JQ353819) and CG-11 (JQ353820) to the closest related strains was 96.01%, 96.29% and 96.19%, respectively (the taxonomic standard for species identification is that a 16S rDNA sequence similarity of less than 97% can be considered a potential new species) (Moreira, Pereira & Thompson, 2011). 19.75% of the clones in the culture-independent method that could represent new taxonomic units were less than 97% similar to the 16S rRNA gene of known bacteria. Furthermore, about 5.75% of the clones had high similarity with uncultured bacteria.

## Conclusions

In this study, we compared the differences between the culture-dependent and culture-independent method in obtaining endophytic bacteria of *P. euphratica*, the endophytic bacteria obtained by the culture-independent method are more abundant in species. Our data indicated that the detection rate of new species in the stem storage liquid samples of *P. euphratica* was high and some isolate strains had biocontrol functions such as plant growth promoting bacteria (PGPB). Therefore, future research should focus on the screening of the dominant endophytic bacteria of *P. euphratica*, biocontrol bacteria with growth promotion and bacteriostasis properties. We should further explore their control effectiveness and action mechanism against leaf rust, grid rust, and trunk rot (ulcer) disease of *P. euphratica*. At the same time, the in-depth exploration of endophytic bacteria resources of *P. euphratica* has great practical application significance for the protection and rejuvenation of the endangered *P. euphratica* forest and the restoration and improvement of the surrounding ecological environment.

## Additional Information and Declarations

### Data Availability

The following information was supplied regarding data availability: The 66 endophytic bacteria strain sequences are available at GenBank: JQ353770 to JQ353835. The 48 OTUs isolated from the bacterial clone library are also available at GenBank: JQ941396 to JQ941443.
